# Supplementary material for: Rules of engagement between αvβ6 integrin and foot-and-mouth disease virus
Source: Nat Commun. 2017 May 23;8:15408. doi: 10.1038/ncomms15408 (PMC5457520; doi:10.1038/ncomms15408)
Supplement: Supplementary Information — Supplementary Figures [file ncomms15408-s1.pdf]

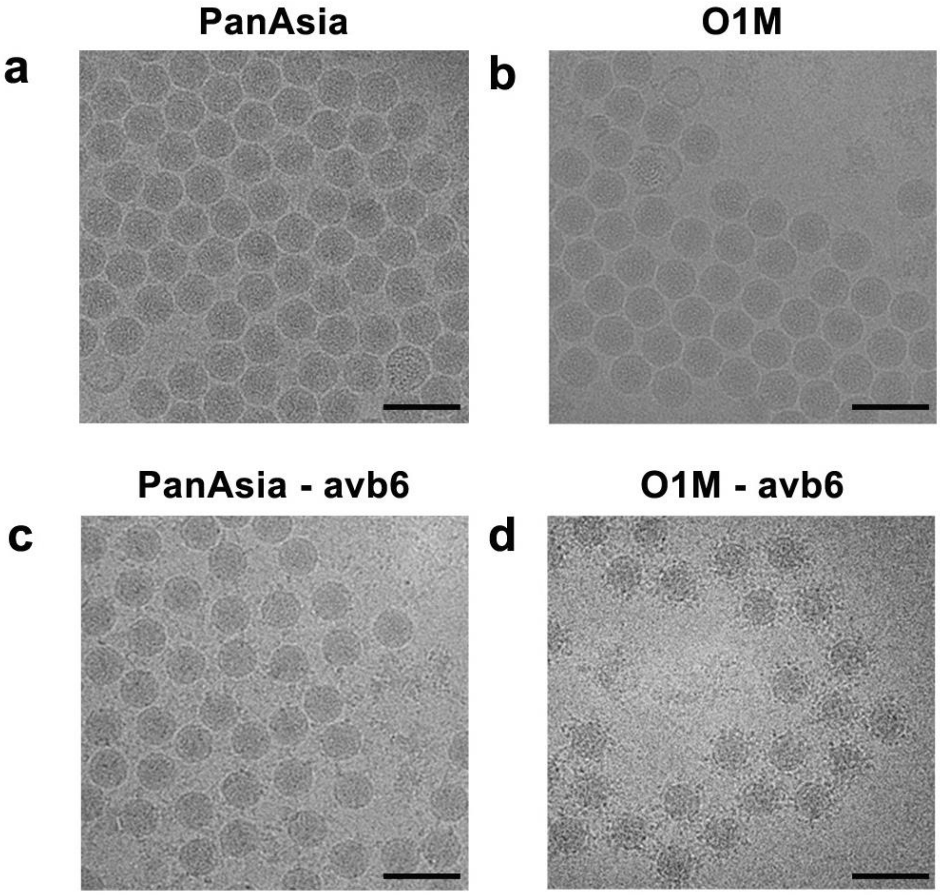

Supplementary Figure 1 | Cryo-electron microscopy of purified FMDV and FMDV-integrin complexes. (a–d) Cryo-EM micrographs showing particles of (a) O Panasia (b) O1M (c) O Panasia decorated with integrin and (d) O1M decorated with integrin. Notice the spiky appearance and larger particle-to-particle distance in the FMDV-integrin complexes when compared to naked FMDV. Scale bar represents 50 nm.

a

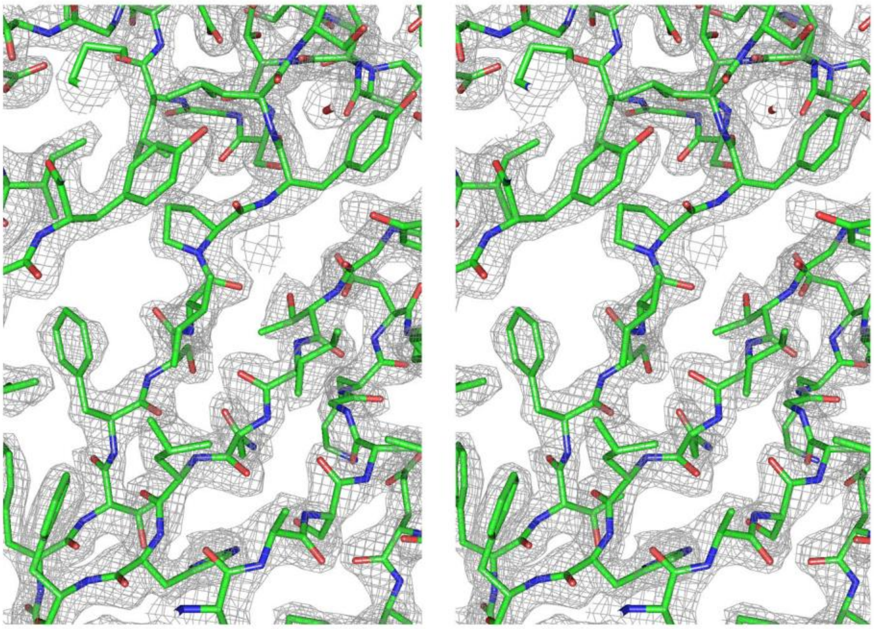

b

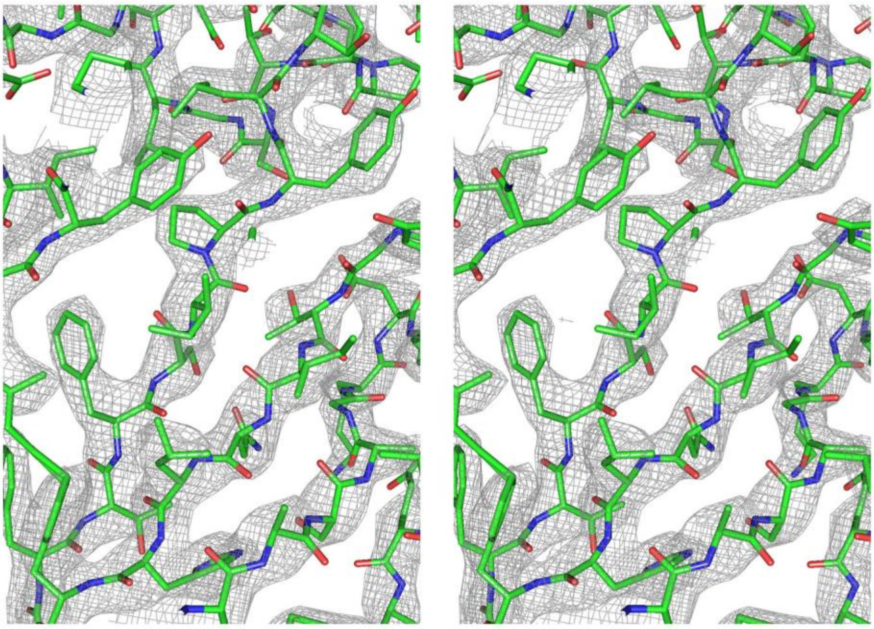

c

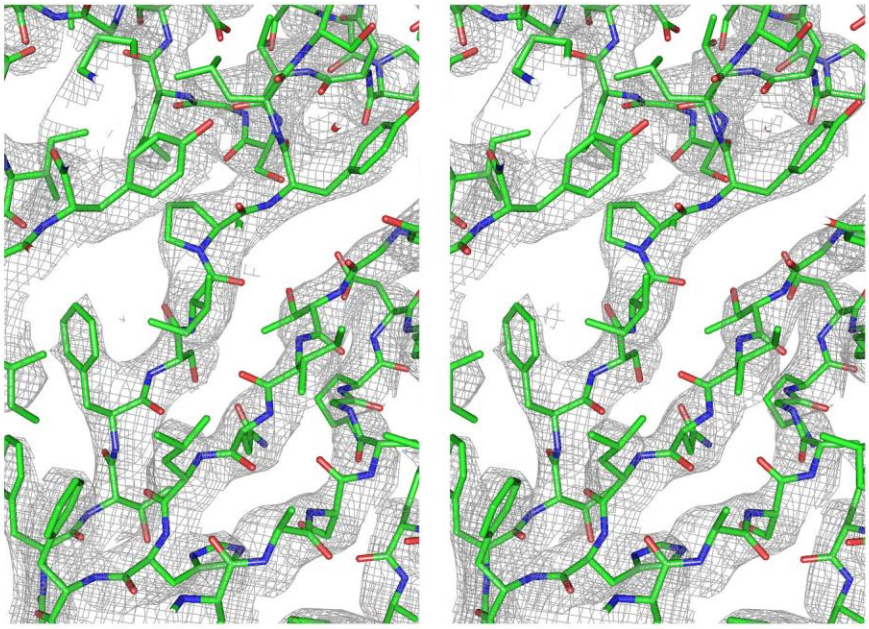

Supplementary Figure 2 | Density maps of FMDV. (a–c) Stereo density maps (grey mesh with the refined atomic model shown as green sticks with atom colouring) are shown for (a) 2.3-Å resolution O PanAsia crystal structure, (b) 3.1-Å resolution O Panasia cryo-EM structure, and (c) 3.5-Å resolution O1M cryo-EM structure.

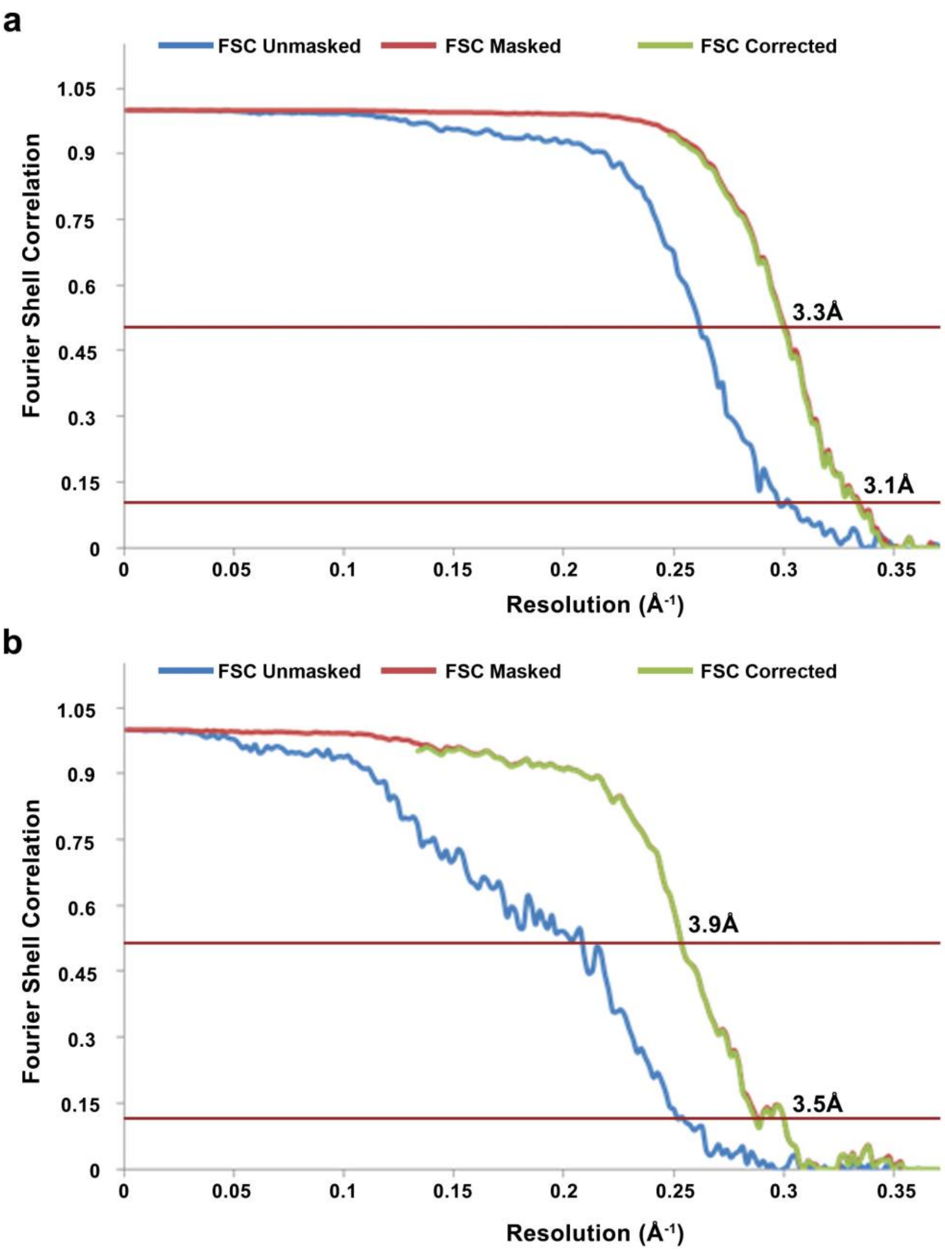

Supplementary Figure 3 | Resolution assessment. (a–b) Fourier shell correlation (FSC) of the final 3D reconstruction obtained using gold-standard refinement using RELION, marked with the resolution corresponding to a FSC of 0.143 are shown for O PanAsia (a) and O1M (b). FSC is plotted before masking (blue), after masking (red), and after correcting for the effect of the mask using phase randomization (green). Notice that the mask had very little effect on the FSC as the red and green curves overlap.

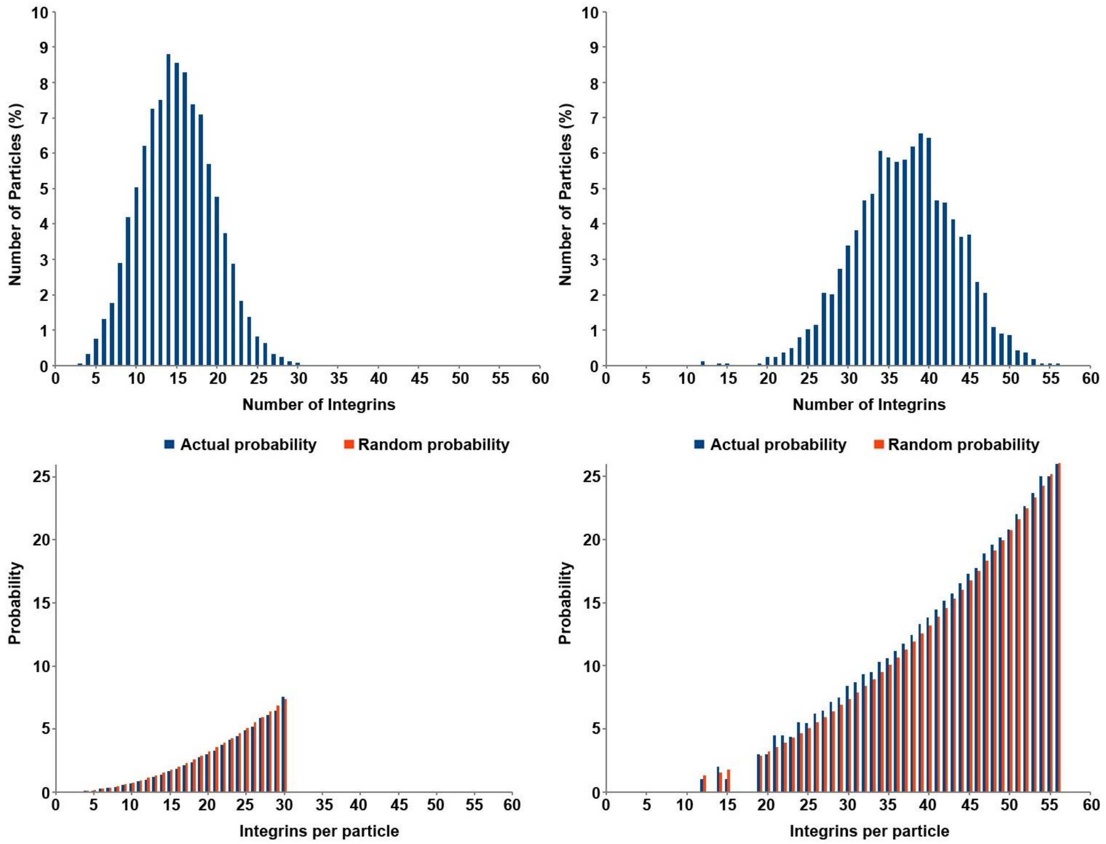

Supplementary Figure 4 | Analysis of integrin occupancy and distribution. (a–b) The occupancy of integrin is shown as a histogram for O PanAsia (a) and O1M (b). **(c–d)** Graphs showing the 2-fold neighbour frequency observed and that expected on the basis of random binding of integrins.

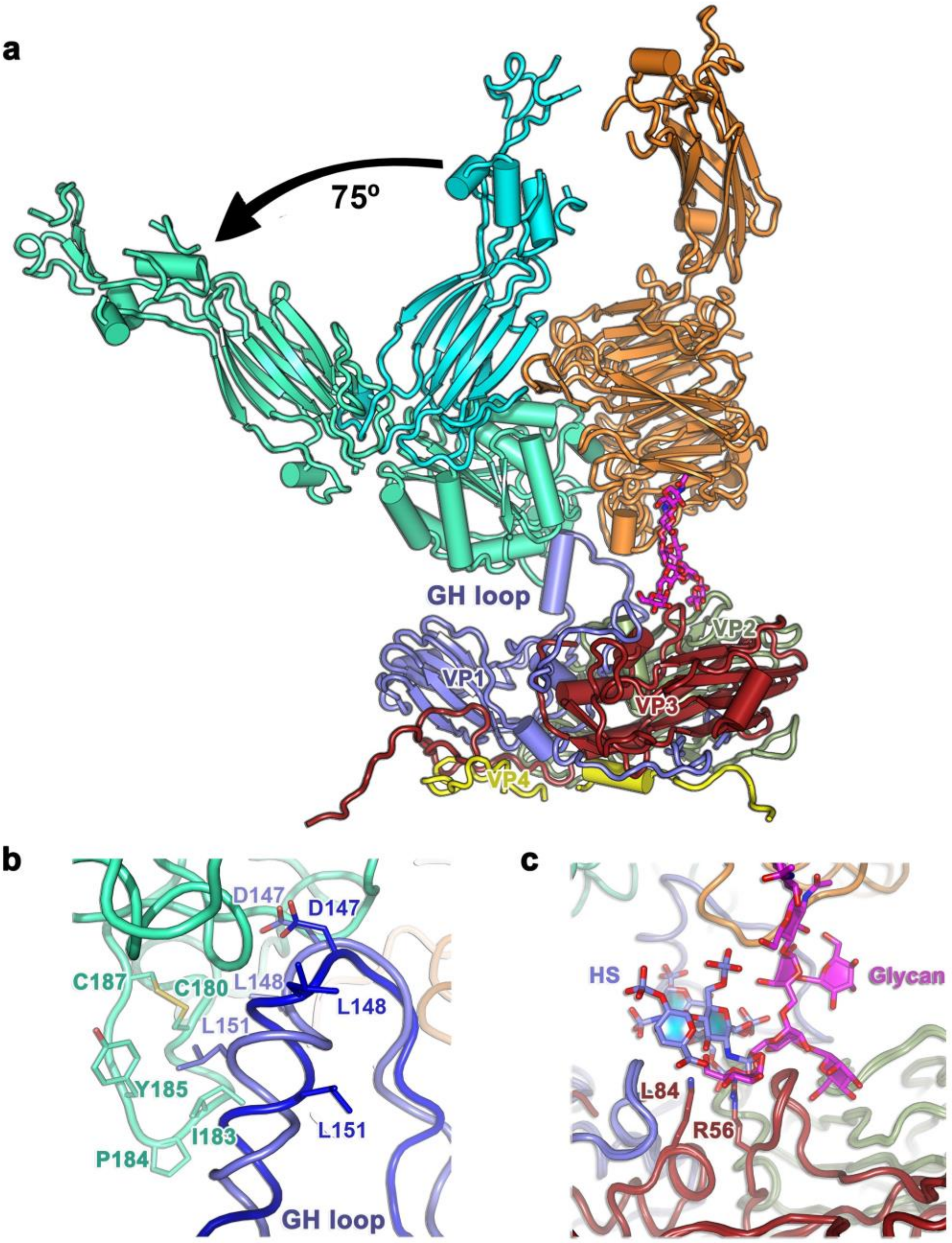

Supplementary Figure 5 | The major binding mode. (a) Binding mode A in cartoon presentation showing the rotation of the integrin 'tail' between open and closed conformations (arrow). **(b)** Comparison of the GH loop without (dark blue) and with integrin (light blue) showing a switch in the backbone conformation between the D of the RGD and the following residue (L148) causing the helix to begin earlier, and rotate ~90° about its long axis to place two leucines (L148 and L151) in the hydrophobic pocket of the integrin. **(c)** Superimposition of FMDV O1BFS-oligosaccharide complex (PDB:1QQP) and the O1M-αvβ6 complex. HS (blue) and Man5GlcNAc2 (magenta) bind to sites close to each other.

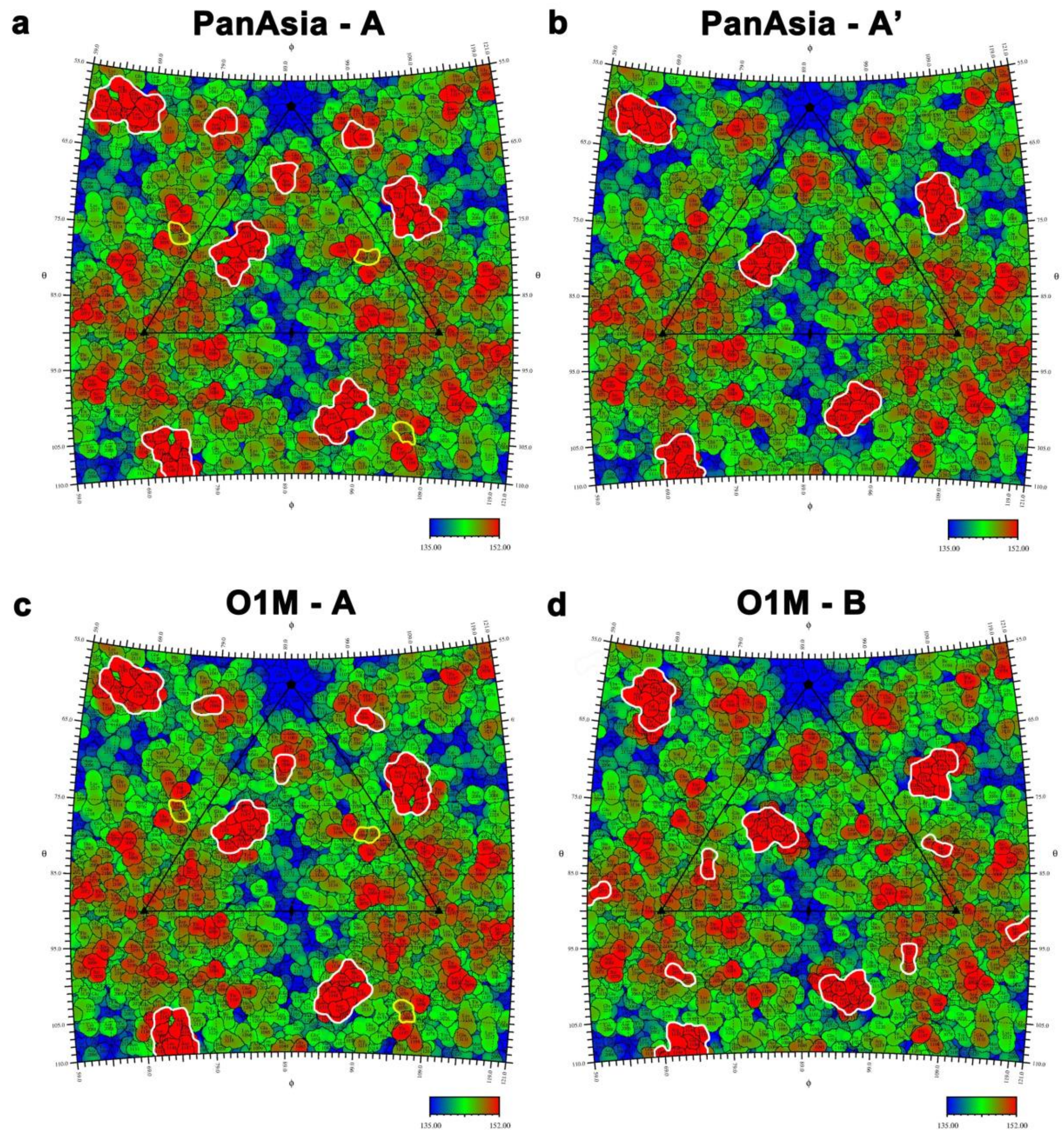

Supplementary Figure 6 | Integrin footprint on the virus surface. (a–d) Roadmap depictions of the icosahedral surface projected onto plane and showing an area larger than one icosahedral face (outlined as a triangle with 2, 3 and 5-fold vertices marked with an ellipse, triangle and a pentagon). The two angles (θ, ϕ) define a vector and further a location on the icosahedron surface. The depictions are radially depth cued from blue (radius = 135 Å) to red (radius = 152 Å). The integrin footprint is outlined in white. Residues are numbered with chain ID preceding residues number, thus VP3 residue 76 is labeled 3076.

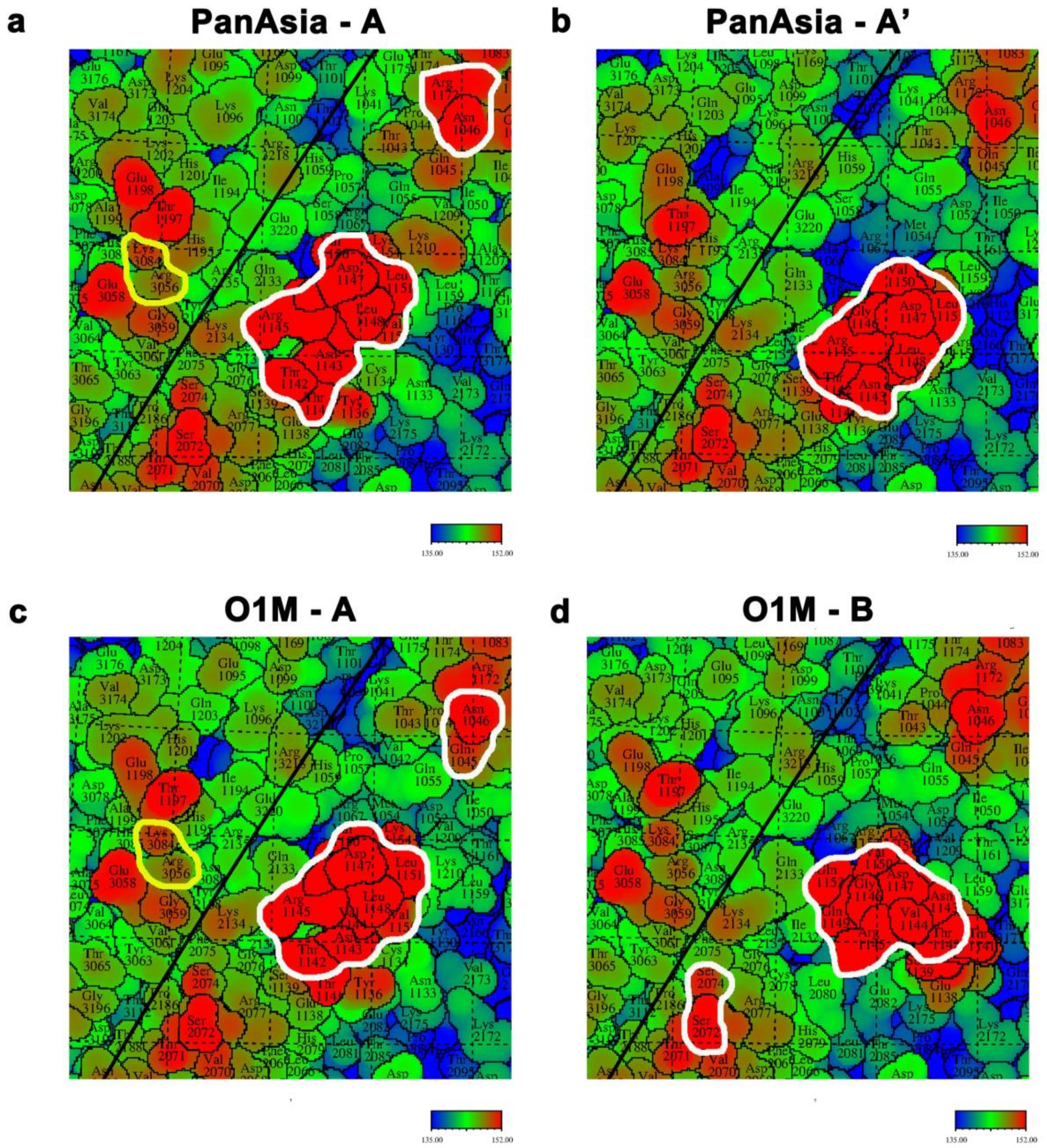

Supplementary Figure 7 | Integrin footprint on the virus surface (enlarged). (a–d) As Supplementary Figure 6 but showing just a single footprint region at a higher magnification.
